# Adherence with post-hospitalization follow-up after pediatric critical illness due to respiratory failure

**DOI:** 10.1186/s12887-024-04888-8

**Published:** 2024-06-26

**Authors:** Lauren M. Yagiela, Marie A. Pfarr, Kathleen Meert, Fola O. Odetola

**Affiliations:** 1 Division of Critical Care, Department of Pediatrics, Children’s Hospital of Michigan, Detroit, MI USA; 2 Department of Pediatrics, Central Michigan University, Mt. Pleasant, MI USA; 3 Department of Pediatrics, Division of Pediatric Hospital Medicine, University of Alabama at Birmingham, Children’s of Alabama, Birmingham, AL USA; 4 Department of Pediatrics and Child Health Evaluation and Research Center, University of Michigan, Ann Arbor, MI USA

**Keywords:** Critical care outcomes, Pediatric intensive care units, Follow-up, Outcome assessment

## Abstract

**Background:**

Adherence with follow-up appointments after a pediatric intensive care unit (PICU) admission is likely a key component in managing post-PICU sequalae. However, prior work on PICU follow-up adherence is limited. The objective of this study is to identify hospitalization characteristics, discharge child health metrics, and follow-up characteristics associated with full adherence with recommended follow-up at a quaternary care center after a PICU admission due to respiratory failure.

**Methods:**

We conducted a retrospective cohort study of patients ≤ 18 years with respiratory failure admitted between 1/2013–12/2014 to a quaternary care PICU. Post-hospitalization full adherence with recommended follow-up in the two years post discharge (1/2013–3/2017) at the quaternary care center was quantified and compared by demographics, baseline child health metrics, hospitalization characteristics, discharge child health metrics, and follow-up characteristics in bivariate and multivariate analyses. Patients were dichotomized into being non-adherent with follow-up (patients who attended less than 100% of recommended appointments at the quaternary care center) and fully adherent (patients who attended 100% of recommended appointments at the quaternary care center).

**Results:**

Of 155 patients alive at hospital discharge, 140 (90.3%) were recommended to follow-up at the quaternary care center. Of the 140 patients with recommended follow-up at the quaternary care center, 32.1% were non-adherent with follow-up and 67.9% were fully adherent. In a multivariable logistic regression model, each additional recommended unique follow-up appointment was associated with lower odds of being fully adherent with follow-up (OR 0.74, 95% CI 0.60–0.91, *p* = 0.005), and each 10% increase in the proportion of appointments scheduled before discharge was associated with higher odds of being fully adherent with follow-up (OR 1.02, 95% CI 1.01–1.03, *p* = 0.004).

**Conclusions:**

After admission for acute respiratory failure, only two-thirds of children were fully adherent with recommended follow-up at a quaternary care center. Our findings suggest that limiting the recommended follow-up to only key essential healthcare providers and working to schedule as many appointments as possible before discharge could improve follow-up adherence. However, a better understanding of the factors that lead to non-adherence with follow-up appointments is needed to inform broader system-level approaches could help improve PICU follow-up adherence.

## Background

The majority of children after a pediatric critical illness require follow-up care after discharge [1–3]. After a pediatric intensive care unit (PICU) admission, up to a third of children have new morbidities, reduced health-related quality of life, and new home care needs [3–11]. Children can also have a traumatic stress response due to a PICU admission, with up to two-thirds of children having posttraumatic stress symptoms after discharge [12–16]. As such, appropriate follow-up with a primary care provider and key specialists is necessary to manage the physical and emotional sequalae from a PICU admission.

A key component of post-hospitalization follow-up is adherence with the recommended follow-up appointments. Adherence with follow-up after a pediatric hospitalization is variable [1, 17–19]. Rates of 100% adherence or full adherence with recommended follow-up range from 53 to 80% for hospitalized children with traumatic brain injury and isolated abdominal injuries, and children after neonatal intensive care unit and PICU admission [1, 17–19]. Prior work has yet to demonstrate a clear pattern of which discharge child health metrics and hospitalization characteristics are associated with non-adherence after pediatric hospitalization [1, 17–19]. Different studies report both increase and decrease in the same factor, such as illness severity or length of stay, as being associated with follow-up non-adherence after pediatric hospitalization [1, 17–19]. Further, limited studies exist that are specific to PICU follow-up adherence [1]. One study, done in the early 2000s by McPherson et al., reported that 65% of patients were fully adherent with recommended PICU follow-up appointments and adherence was better with primary care appointments than specialty appointments [1].

While studies have begun to describe follow-up adherence after pediatric hospitalization, few studies have focused on follow-up adherence after a PICU admission. Further, the literature on factors associated with poor follow-up adherence after a PICU admission is limited. Specifically, we lack robust data on how hospitalization characteristics, discharge child health metrics, and follow-up characteristics impact follow-up adherence. Understanding how hospitalization characteristics, discharge child health metrics, and follow-up characteristics impact follow-up adherence could inform the development of a hospital system-based approach to improve PICU follow-up adherence and post-PICU care delivery.

In this study, we evaluated post-hospitalization follow-up full adherence after a critical illness secondary to respiratory failure. Respiratory failure is the most common primary diagnosis in the PICU and as such, patients with respiratory failure represent a substantial majority of children requiring PICU follow-up. We hypothesize that key hospitalization characteristics, discharge child health metrics, and follow-up characteristics will be associated with adherence with follow-up. The study objectives are, in patients after pediatric critical illness due to respiratory failure, to: (1) report rates of post-hospitalization follow-up full adherence at a quaternary care center; and (2) evaluate the association between hospitalization characteristics, discharge child health metrics, and follow-up characteristics with full adherence to recommended follow-up appointments.

## Methods

### Study design and setting

This is a retrospective cohort study of children ≤ 18 years admitted to a quaternary PICU from 1/1/2013–12/31/2014 with respiratory failure secondary to an intrapulmonary process. Post-hospitalization full adherence with follow-up in the two years post discharge (1/2013–3/2017) at the quaternary care center was quantified. While most follow-up was expected to occur a few months after discharge, a two-year follow-up period was used to capture appointments further out from discharge. The study setting was a quaternary PICU with 1200 admission per year with capacity to provide specialized intensive care including extracorporeal membrane oxygenation (ECMO) and continuous renal replacement therapy (CRRT). This study was a planned secondary analysis of data collected during a prior study of outcomes and healthcare utilization after a PICU admission for respiratory failure [2, 3]. All data for this analysis was collected at the time of the original study. At the time of data collection, the study institution did not have a PICU follow-up clinic or outpatient complex care program. The study institution did have resident assistants, staff members assigned to help the clinical team with administrative tasks including scheduling follow-up appointments prior to a child’s discharge. This study was approved by the Institutional Review Board (IRB, IRB #HUM00100246) of the University of Michigan Medical School in accordance with the Declaration of Helsinki. Informed consent was waived by the IRB as this was a retrospective analysis of already existing data.

## Inclusion / exclusion criteria and data collection

We included children ≤ 18 years admitted to a quaternary PICU from 1/1/2013–12/31/2014 with respiratory failure secondary to an intrapulmonary process. These patients were identified using an institutional Virtual PICU System (VPS) database. The VPS database was maintained by designated trained data entry specialist who entered information upon admission including patient demographics, primary diagnosis, and existing comorbid conditions using International Classification of Diseases-Clinical Modification version 9 (ICD-9) codes. To identify patients, a VPS query identified all patients from 1/1/2013–12/31/2014 with a respiratory system-associated ICD-9 codes. Then, patients with a primary diagnosis of respiratory failure due to an intrapulmonary process were identified through a manual review of the medical chart using the following definitions. Respiratory failure was defined as the receipt of noninvasive or invasive positive pressure ventilation, as described in our prior work [2, 3]. An intrapulmonary process was defined as a pulmonary illness including pneumonia, asthma, acute lung injury, and bronchiolitis, as described in our prior work [2, 3]. We excluded patients born during the index hospitalization, only intubated for airway protection, who died during the hospital admission, or who were transferred to another institution prior to discharge home.

Patient-level data, including demographics, comorbid conditions, and hospitalization characteristics, were obtained from the institutional VPS database. Additional patient-level data on Functional Status Scale (FSS) score [20], home care needs, and follow-up appointments were obtained through medical chart review. Data was collected using two excel spreadsheets, in accordance with IRB regulations. One spreadsheet included patient identifiers linked with a study ID number with no study variables. The other spreadsheet contained a study ID number and all the study variables. After data collection, the data was checked for errors and cleaned after which the spreadsheet with patient identifiers was destroyed in keeping with data security measures at the time of the study.

## Study variables

The following categories of variables were collected: demographics, baseline child health metrics, hospitalization characteristics, discharge child health metrics, and follow-up appointment characteristics. Demographic data included age and gender. Baseline child health metrics included comorbid conditions and FSS score on admission. Comorbid conditions were identified with the use of ICD-9 diagnosis codes for complex chronic conditions as described by Feudtner et al. [21]. The FSS assesses a child’s functional status in six domains (mental, sensory, communication, motor function, feeding, and respiratory) [20]. The FSS score was manually calculated after review of the medical chart [20]. The FSS scores range from 6 to 30 [20]. A score of six indicates normal function, with higher scores indicating worse function [20]. Hospitalization characteristics, as described previously [2, 3], included: (1) severity of illness measured using the Pediatric Risk of Mortality (PRISM) version III score-derived probability of mortality [22]; (2) type and duration of positive pressure ventilation; (3) adjunctive therapies (vasoactive medication use, ECMO use [venovenous and venoarterial]; (4) CRRT use; and (5) length of stay (LOS) in the PICU and hospital. Discharge child health metrics included discharge FSS score, new morbidity assessment, new medical devices, and home care equipment. As described by Pollack et al. [20], new morbidity was defined as a change in FSS score of greater than or equal to 3 at hospital discharge relative to pre-admission baseline. Home care needs included new medical devices (e.g. tracheostomy, gastrostomy tube) and home care equipment (e.g. mechanical ventilators, feeding supplies), as described in our prior work [2, 3].

Recommend follow-up appointments were all primary care or specialty care appointments that patients were recommended to follow-up with in the discharge summary. Appointments were considered scheduled if the appointment date and time were listed on the discharge summary. Scheduling data were captured for follow-up at both community care centers and the quaternary care center in the two years after discharge. Attendance at follow-up was collected only for appointments at the quaternary care center. The appointment was considered attended if the medical record at the quaternary care center had a note documenting an in-person patient encounter with a healthcare provider. To allow for inclusion of rescheduled appointments in the captured attendance data, appointments were considered to have been attended if the visit occurred within 4 months of the date specified in the discharge summary document or if the visit occurred within 4 months of hospital discharge if no date was noted in the discharge summary document. Patients were dichotomized into being non-adherent with follow-up (patients who attended less than 100% of recommended appointments at the quaternary care center) and fully adherent with follow-up (patients who attended 100% of recommended appointments at the quaternary care center).

## Statistical analysis

Data analysis was done using STATA version 14 (STATA, College Station, TX). Continuous data are presented as medians and interquartile ranges, and categorical data as frequencies and percentages.

Three separate analyses were completed:

1. Descriptive statistics on demographics and proportion of appointments scheduled before discharge (number of appointments scheduled / number of appointments recommended) for all patients were completed. Descriptive statistics on proportion of appointments attended (number of appointments attended at quaternary care center / number of appointments recommended at quaternary care center) and proportion of patients with full adherence (patients who attended 100% of recommended appointments at the quaternary care center) were completed for patients with follow-up appointments at the quaternary care center. The proportion of patients with full adherence was calculated for all appointments (all primary care and specialty care appointments combined), primary care appointments, all specialty care appointments, and the top four specialties (Pulmonary, Otolaryngology, Cardiology, and Physical Therapy/Occupational Therapy) that patients were recommended to follow-up with, as identified in prior work with this dataset [3].
2. Bivariate analysis compared patients who were non-adherent with follow-up appointments at the quaternary care center with those who were fully adherent with follow-up appointments at the quaternary care center with respect to demographics, baseline child health metrics, hospitalization characteristics, discharge child health metrics, and follow-up characteristics. Bivariate comparisons were made using chi-square test for categorical variables and Wilcoxon Rank Sum test for continuous variables.
3. Multivariable logistic regression models were fit to compare the impact of demographics, baseline child health metrics, hospitalization characteristics, discharge child health metrics, and follow-up appointment characteristics on full adherence with follow-up (comparing non-adherent patients with follow-up at the quaternary care center with those who were fully adherent with follow-up at the quaternary care center). Independent variables were included in the model through two processes: 1) if they were associated (*p* < 0.2) with the outcome variables in bivariate analysis, and 2) if they were deemed clinically relevant, including patient age, gender, and severity of illness. This multivariable model was additionally used to calculate predicted probabilities of full adherence based on individual covariates, with an assumption that follow up clinic visits were independent events with a linear continuous distribution.

## Results

### Admission health, hospitalization characteristics, discharge health

All 155 discharged patients had follow-up appointments recommended after discharge, with 140 of these having recommended follow-up appointments at the quaternary care center. Table 1 summarizes the patient characteristics and hospitalization details for all 155 patients. The median age was 2.1 years (IQR, 0.7–10.6 years) and 65 (41.9%) were female. Median admission FSS score was 7 (IQR, 6–11) and 61 (39.4%) patients had a comorbid condition at baseline. The median PICU LOS was 8 days (IQR, 4–15) while the median hospital LOS was 13 days (IQR, 8–23 days). At hospital discharge, 14 (9%) patients had a new morbidity and the median discharge FSS score was 9 (IQR 6–12).

**Table 1:** Patient and hospitalization characteristics and bivariate analysis of patients who were non-adherent with follow-up and adherent after PICU admission for respiratory failure

	**All Discharge Patients (*n*** **= 155)**	**Discharged Patients with Follow-up at the Quaternary Care Center (*n*** **= 140)**		**P**^**b**^
		**Non-Adherent with Follow-up (*n*** **= 45)**	**Fully Adherent with Follow-up (*n*** **= 95)**	
**Demographics and Baseline Child Health Metrics**				
Age (years), median (IQR)	2.1 (0.7–10.6)	1.6 (0.8–12.8)	2.8 (0.8–10.4)	0.945
Gender, n (%)				0.088
Female	65 (41.9)	14 (31.1)	44 (46.3)	
Male	90 (58.1)	31 (68.9)	51 (53.7)	
Comorbid Condition at Admission, n (%)	61 (39.4)	22 (48.9)	37 (39.0)	0.266
Neurologic	24 (15.5)			
Genetic	18 (11.6)			
Cardiac	16 (10.3)			
Respiratory	11 (7.1)			
FSS at Admission, median (IQR)	7 (6–11)	8 (6–13)	7 (6–12)	0.637
**Hospitalization Characteristics**				
PRISM Calculated Probability of Death, n (%)				0.721
< 5% probability of death	120 (77.4)	33 (73.3)	73 (76.8)	
5–30% probability of death	31 (20.0)	10 (22.2)	20 (21.1)	
> 30% probability of death	4 (2.6)	2 (4.4)	2 (2.1)	
Highest Level of Ventilatory Support				0.017
Noninvasive	23 (14.8)	10 (22.2)	12 (12.6)	
Conventional	127 (81.9)	31 (68.9)	82 (86.3)	
Oscillator	5 (3.2)	4 (8.9)	1 (1.1)	
Duration of Positive Pressure Ventilation (days), median (IQR)	5.8 (2.9–11.7)	6.7 (3.5–15.1)	6.2 (2.9–11.7)	0.713
Vasopressors^a^, n (%)	52 (33.6)	15 (33.3)	33 (34.7)	0.870
ECMO, n (%)	11 (7.1)	4 (8.9)	7 (7.4)	0.755
CRRT, n (%)	7 (4.5)	5 (11.1)	2 (2.1)	0.022
LOS PICU, median (IQR)	8 (4–15)	9 (4–18)	9 (4–15)	0.871
LOS hospital median (IQR)	13 (8–23)	16 (9–30)	13 (8–22)	0.135
**Discharge Child Health Metrics**				
FSS at Discharge, median (IQR)	9 (6–12)	10 (7–13)	9 (6–12)	0.268
New Morbidity, n (%)	14 (9.0)	6 (13.3)	8 (8.4)	0.366
New Medical Device, n (%)	24 (15.5)	10 (22.2)	14 (14.7)	0.272
New Medical Equipment, n (%)	43 (27.7)	19 (42.2)	24 (25.3)	0.042
**Follow-Up Characteristics**				
Total Number of Follow-up Appointments, median (IQR)	3 (2–5)	5 (3–6)	3 (2–4)	0.003
Percentage Scheduled Prior to Discharge, median (IQR)	80% (40%-100%)	100% (40%-100%)	100 (100%-100%)	< 0.001

*IQR*  interquartile range, *FSS*  Functional status scale, score 6–30 with higher number indicative of worse function, *ECMO*  Extracorporeal membrane oxygenation, *CRRT*  Continuous renal replacement therapy, *PRISM*-III  Pediatric Risk of Mortality Version 3, *PICU*  Pediatric intensive care unit

^a^Vasoactive medications include epinephrine, norepinephrine, vasopressin, dopamine, and milrinone

^b^p for comparison between groups non-adherent with follow-up and adherent with follow-up using Wilcoxon Rank Sum for continuous variables and chi-square for categorical variable

## Post-hospitalization follow-up

**Fig. 1:**
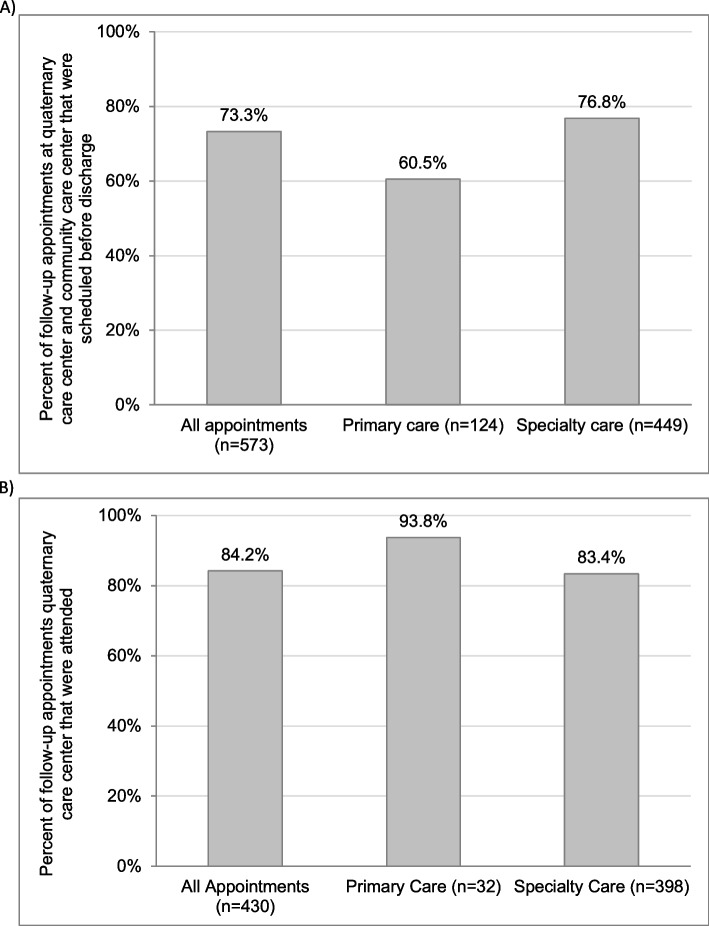
**A** Percent of appointments scheduled before discharge by appointment type (*n* = 155 patients). **B** Percent of appointments attended by appointment type (*n* = 140 patients)

The 155 patients had a total of 573 recommended follow-up appointments at either the quaternary care center or community care center (Fig. 1A). Of these, 449 were specialty care appointments and 124 were primary care appointments. The majority of appointments (420/573, 73.3%) were scheduled prior to discharge (Fig. 1A).

Regarding appointments at the quaternary care center (Fig. 1B), 140 patients had a total of 430 appointments recommended at the quaternary care center. The majority of follow-up appointments at the quaternary care center were attended (*n* = 362/430, 84.2%) (Fig. 1B).

**Fig. 2:**
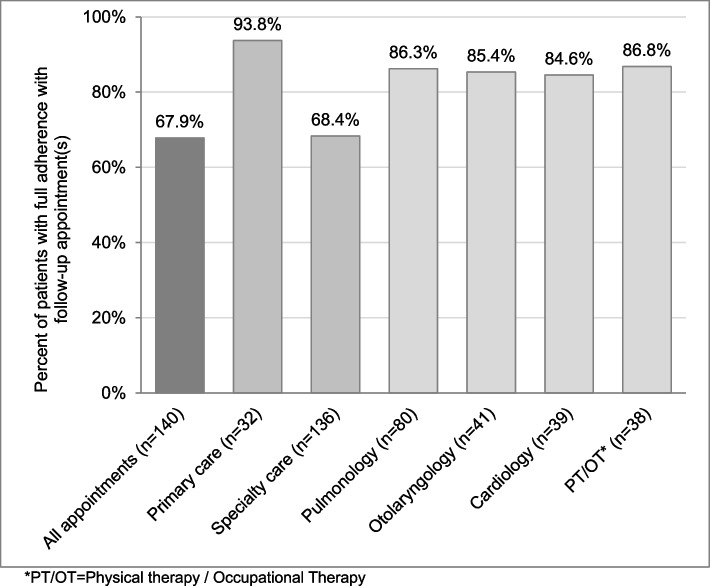
Percent of patients with full adherence with recommended follow-up appointment(s) for: all appointments (all primary care and specialty care appointments), primary care appointments, specialty care appointments and the top four specialties (Pulmonary, Otolaryngology, Cardiology, and Physical Therapy/Occupational Therapy) that patients were recommended to follow-up with, as identified in prior work using the same dataset [3]

Regarding full adherence with follow-up by patient at the quaternary care center overall (Fig. 2), there were 95 patients (*n* = 95/140, 67.9%) who were fully adherent with follow-up (attended 100% of recommended follow-up at the quaternary care center) and 45 patients (*n* = 45/140, 32.1%) who were non-adherent with follow-up (attended less than 100% of recommended follow-up at the quaternary care center). Ninety-three percent (*n* = 30/32) of patients were fully adherent with recommended primary care follow-up at the quaternary care center and 68.4% (*n* = 93/136) were fully adherent with recommended specialty care follow-up (Fig. 2). Full adherence with the top four specialties that participants were recommended to follow-up with ranged from 84.6% to 86.3% (Fig. 2).

## Factors associated with full adherence with follow-up appointments

The bivariate analyses assessing for the association between demographics, baseline child health metrics, hospitalization characteristics, discharge child health metrics, and follow-up characteristics with full adherence with recommended follow-up are listed in Table 1. In bivariate analysis, patients who were fully adherent were more likely to have received conventional ventilation, less likely to have received CRRT, and less likely to have received new medical equipment (Table 1). Fully adherent patients also had fewer recommended follow-up appointments and had a higher percentage follow-up appointment scheduled before discharge (Table 1).

**Fig. 3:**
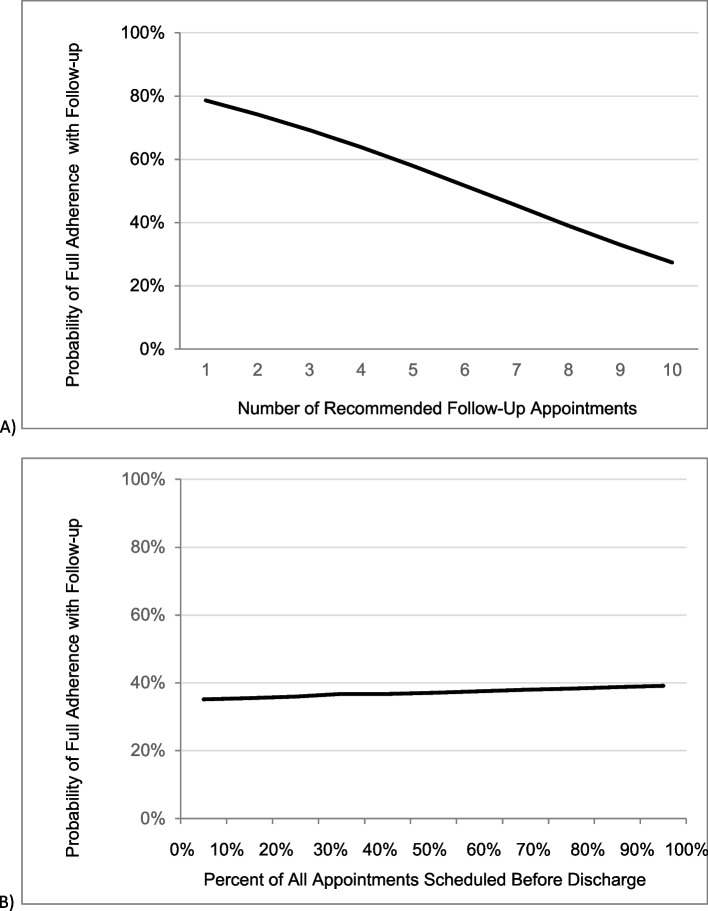
**A** Probability of full adherence with follow-up as the number of recommended follow-up appointments increases (**B**) Probability of full adherence with follow-up as the percent of all appointments scheduled before discharge increases

The results of the multivariable logistic regression model are in Table 2. A higher number of follow-up appointments was associated with lower odds of being fully adherent with follow-up (OR 0.74, 95% CI 0.60–0.91, *p* = 0.005). Specifically, for each additional recommended unique appointment, the odds ratio of being fully adherent with follow-up was 0.74. As the number of recommended appointments after discharge increased from 1 to 10 appointments, the multivariable model-generated predicted probability of being fully adherent with follow-up decreased from 78 to 27% (Fig. 3a). Additionally, a higher percent of appointments scheduled before discharge was associated with higher odds of being fully adherent with follow-up (OR 1.02, 95% CI 1.01–1.03, *p* = 0.004). Specifically, for each 10% increase in the proportion of appointments scheduled before discharge, the odds of being fully adherent were 1.02. As the percentage of follow-up appointments scheduled before discharge increased from 0 to 100%, the multivariable model-generated predicted probability of being fully adherent with follow-up increased from 35 to 39% (Fig. 3b).

**Table 2:** Multivariable model of factors associated with being 100% compliant with follow-up after PICU admission for respiratory failure

	**Odds Ratio**	**95% Confidence Interval**	**P**
Age	1.00	0.99-1.01	0.72
Gender			
Female	Reference		
Male	0.48	0.19-1.17	0.108
PRISM-III calculated percent probability of death	1.01	0.97-1.04	0.712
Highest level of ventilatory support			
Noninvasive	Reference		
Conventional	11.51	0.91-145.80	0.059
Oscillator	4.96	0.34-71.37	0.239
Receipt of CRRT	0.57	0.06-5.04	0.615
LOS hospital in days	0.99	0.98-1.01	0.653
Receipt of new medical equipment	0.64	0.23-1.7	0.380
Number of Follow-up Appointments^a^	0.74	0.60-0.91	0.005
Percent (in 10% increments) of appointments scheduled prior to discharge^b^	1.02	1.01-1.03	0.004

*CRRT* Continuous renal replacement therapy, *PRISM-III* Pediatric Risk of Mortality Version 3, *PICU* Pediatric intensive care unit, *LOS* Length of stay

^a^e.g. for each additional appointment, the odds of being fully compliant with follow-up was 0.75)

^b^e.g. for each 10% increase in the percent of appointments scheduled before discharge, the odds of being fully compliant were 1.22

## Discussion

Our study findings describe the hospitalization characteristics, discharge child health metrics, and follow-up characteristics associated with full adherence with follow-up recommendations after pediatric critical illness for respiratory failure. Specifically, three quarters of appointments were scheduled before discharge and only 68% of children were fully adherent or attended all recommended follow-up appointments at the quaternary care center. Further, more patients were fully adherent with primary care follow-up than specialty care follow-up. We found in a multivariable model that as the number of appointments recommended at discharge increased, a child had lower odds of being fully adherent with recommended appointments after discharge. In contrast, we found that as the percentage of appointments scheduled prior to discharge increased, a child had higher odds of being fully adherent with recommended follow-up appointments. Other variables including demographics, baseline and discharge child health metrics, or hospitalization characteristics were not significantly associated with follow-up full adherence.

Our findings and prior literature suggest that adherence with follow-up after a PICU admission could be improved by reducing potential systems-level barriers to follow-up. One potential barrier to full adherence with follow-up is the burden of having multiple follow-up appointments [1, 19]. In our study, we found that as the number of appointments recommended at discharge increased, a child had a lower odds of being fully adherent with follow-up. McPherson et al. [1] also found that non-adherence with follow-up after a PICU admission was associated with higher numbers of follow-up appointments recommended at discharge. Spaw et al. [19], found that children recommended to follow-up with more than one department were less likely to be fully adherent with follow-up appointments after a traumatic brain injury admission. Another potential barrier to full adherence with follow-up after a PICU admission is parents needing to schedule appointments after discharge. Our study found that as the percentage of appointments scheduled prior to discharge increased, the child had higher odds of being fully adherent with recommended follow-up appointments. McPherson et al. [1] found that parents cited communication errors such as difficulty with scheduling appointments and lack of phone numbers for specialists as a reason for non-adherence with PICU follow-up.

Our findings and prior literature suggest a structured follow-up plan that engages system-level approaches could help improve adherence with PICU follow-up appointments. In the structured follow-up plan, prior literature suggests six key areas to address when planning and supporting parents to attend follow-up appointments [23]. First, the team discharging the child should limit to the extent possible the number of follow-up appointments, given our findings and prior literature that more follow-up appointments are associated with less adherence [1, 19, 23]. Second, follow-up appointments and any transportation assistance a family needs for that appointment should be scheduled, with family input on their availability, before discharge [1, 23]. Third, multiple appointments should be scheduled for the same day or follow-up in a multidisciplinary PICU follow-up clinic should be arranged. A PICU multidisciplinary follow-up clinic would allow a child to see multiple specialists on the same day [24–31]. The utility of, and type of specialist needed for, a multidisciplinary clinic could be informed by a prior analysis of the dataset used in this current study [3]. In our prior analysis, we found that the majority of patients (86.5%) were referred to two or more follow-up appointments, with a range of 1 to 10 follow-up appointments [3]. The most common healthcare providers patients with respiratory failure were recommended to follow up with were primary care (80%), pulmonology (52.9%), physical and occupational therapy (51.6%), otolaryngology (26.5%), and cardiology (25.8%) [3]. Multidisciplinary clinics in pediatrics have been shown to improve care coordination, reduce medical travel time, improve parental understanding of a child’s condition, improve child health outcomes, and reduce ED visits [29–31]. Combining follow-up with multiple specialties may reduce the burden of follow-up appointments on the parents, as this reduces the number of days a parent would miss work, and need sibling childcare and transportation. Fourth, provide sibling childcare services in specialty clinics, which reduces the burden on parents to arrange sibling childcare for appointments. Fifth, remind parents of appointments using text messages, as this has been shown to increase attendance at follow-up appointments [32]. Finally, work to engage primary care providers or care coordinators to monitor follow-up attendance and help support parents [33]. Care coordinators have been shown in multidisciplinary clinics to improve adherence with attendance [34]. Creating a multidisciplinary approach that addresses system-level solutions is a unique and innovative method to help improve adherence with PICU follow-up.

This study has a few limitations. First, this is a single-center study. Additionally, we were only able to capture appointment adherence at the quaternary care center and as such, do not have an assessment of adherence with community-based follow up appointments. Further, adherence rates could have been affected by resolution of medical issues after discharge. For instance, a patient could have been recommended to follow-up with a specialist and then upon initial follow-up with their primary care provider, the medical issue they needed to see the specialist for could have resolved. In this study, we chose to focus on hospitalization characteristics, discharge child health metrics, and follow-up characteristics. Further, our data collection methods, whereby after data collection patient identifiers were destroyed, limited our ability to collect additional variables after our initial analysis. While the data presented in the manuscript furthers our understanding of PICU follow-up adherence, a more robust understanding of PICU follow-up requires that future work include analysis of individual and family-level variables, variables related to social determinants of health, and additional variables on follow-up appointment characteristics. Individual and family-level variables that future work should explore include race, ethnicity, English literacy, medical literacy, primary language, parent work flexibility, and availability of childcare [35]. Social determinants of health variables that future work should analyze include socioeconomic status, housing and food security, financial security, and transportation access [35]. Additional follow-up appointment variables that should be assessed in future work include discharge destination (home vs rehabilitation facility), the distance to the hospital, the median time from hospital discharge to follow-up appointments, which specialty providers patients had previously seen, the timing of follow-up after discharge, and how many subjects missed just one appointment. While the data reported in this manuscript are from 2013–2017, these findings are an important step in understanding PICU follow-up adherence. The last study that examined PICU follow-up adherence was published in 2002 by McPherson et al. [1] and this current study builds on this prior study. Future studies on, and interventions for, PICU follow-up adherence can build on the findings and limitations reported in our study. Additional investigation into PICU follow-up adherence is key to moving this area of research forward.

## Conclusions

After admission for acute respiratory failure, only two-thirds of children were fully adherent with recommended follow-up at a quaternary care center. Our findings suggest that limiting the recommended follow-up to only key essential healthcare providers and working to schedule as many appointments as possible before discharge could improve follow-up adherence. However, a better understanding of the factors that lead to non-adherence with follow-up appointments is needed to inform broader system-level approaches could help improve PICU follow-up adherence.

## Data Availability

The dataset supporting the conclusions of this article is available upon request to the corresponding author (Lauren Yagiela, yagie1lm@cmich.edu).
